# A Case of Bullous Sweet’s Syndrome Associated With Esophageal Adenocarcinoma

**DOI:** 10.7759/cureus.52954

**Published:** 2024-01-25

**Authors:** Adriana G Bagos-Estevez, Sarah Moore, Leslie Turner, Brooke Baldwin

**Affiliations:** 1 Dermatology, University of South Florida Morsani College of Medicine, Tampa, USA; 2 Dermatopathology, James A. Haley Veterans' Hospital, Tampa, USA; 3 Dermatology, James A. Haley Veterans' Hospital, Tampa, USA

**Keywords:** esophageal adenocarcinoma, sweet’s syndrome, neutrophilic dermatosis, malignancy, bullous

## Abstract

Sweet’s syndrome (SS), or acute febrile neutrophilic dermatosis, characteristically presents with fever, dermal neutrophilic infiltrates, and neutrophilia. It typically manifests as tender erythematous plaques; however, various variants are documented, including bullous. Malignancy-associated Sweet’s syndrome (MASS) can present as a paraneoplastic syndrome in those with established cancers or with undiagnosed malignancies.

We present a 72-year-old male with a three-day history of a progressive bullous, erythematous papular rash starting on his right forearm and spreading to his extremities, trunk, palms, and soles. It was mildly pruritic but nontender. He had no recent febrile illnesses. On examination, the rash was violaceous with tense bullae overlying edematous targetoid papules coalescing into plaques. Histopathologic analysis of punch biopsies from his abdomen and thigh demonstrated dense inflammatory infiltrates of neutrophils, eosinophils, histiocytes, and lymphocytes, suggestive of neutrophilic dermatosis, or Sweet’s syndrome. He was treated with prednisone 1 mg/kg with improvement in his cutaneous symptoms, and a malignancy workup was initiated. Blood work showed elevated free kappa, lambda light chains, lactate dehydrogenase (LDH), and C-reactive protein (CRP) levels. A positron emission tomography (PET) scan revealed lesions in the esophagus and kidney. He was referred to Heme/Onc, GI, and Urology. He was diagnosed with esophageal adenocarcinoma stage IIb and a renal mass. He has since completed neoadjuvant chemotherapy and radiation, is s/p robotic Ivor-Lewis esophagectomy with no evidence of residual carcinoma on pathology, and is undergoing surveillance with imaging every three months for his renal mass. This case highlights the importance of rapid identification of MASS and the impact dermatologists can make in getting these patients the potentially lifesaving care they need.

## Introduction

Sweet’s syndrome (SS), also known as acute febrile neutrophilic dermatosis, characteristically presents with fever, dermal neutrophilic infiltrates, and neutrophilia [1]. Typical cutaneous manifestations include tender, erythematous, edematous plaques; however, several variants have been documented, including localized neutrophilic dermatosis of the dorsal hands and bullous, subcutaneous, cellulitic, and necrotizing lesions [2]. Malignancy-associated Sweet’s syndrome (MASS) can present as a paraneoplastic syndrome in a patient with an established cancer diagnosis or those with an underlying undiagnosed malignancy [1]. The absence of fever and neutrophilia has been documented in biopsy-confirmed cases of MASS [3].

## Case presentation

**Figure 1:**
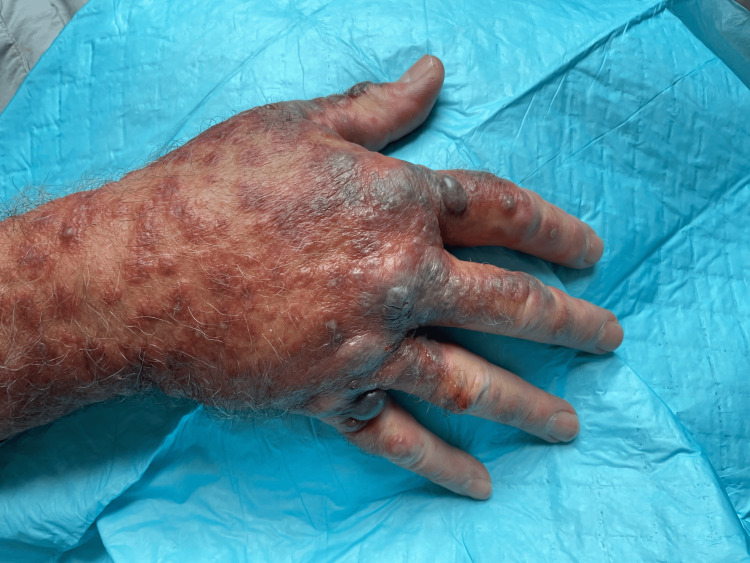
Image of the right distal forearm and dorsal hand. Seen here is an erythematous, papular, and violaceous rash with tense bullae overlying edematous targetoid papules coalescing into plaques.

**Figure 2:**
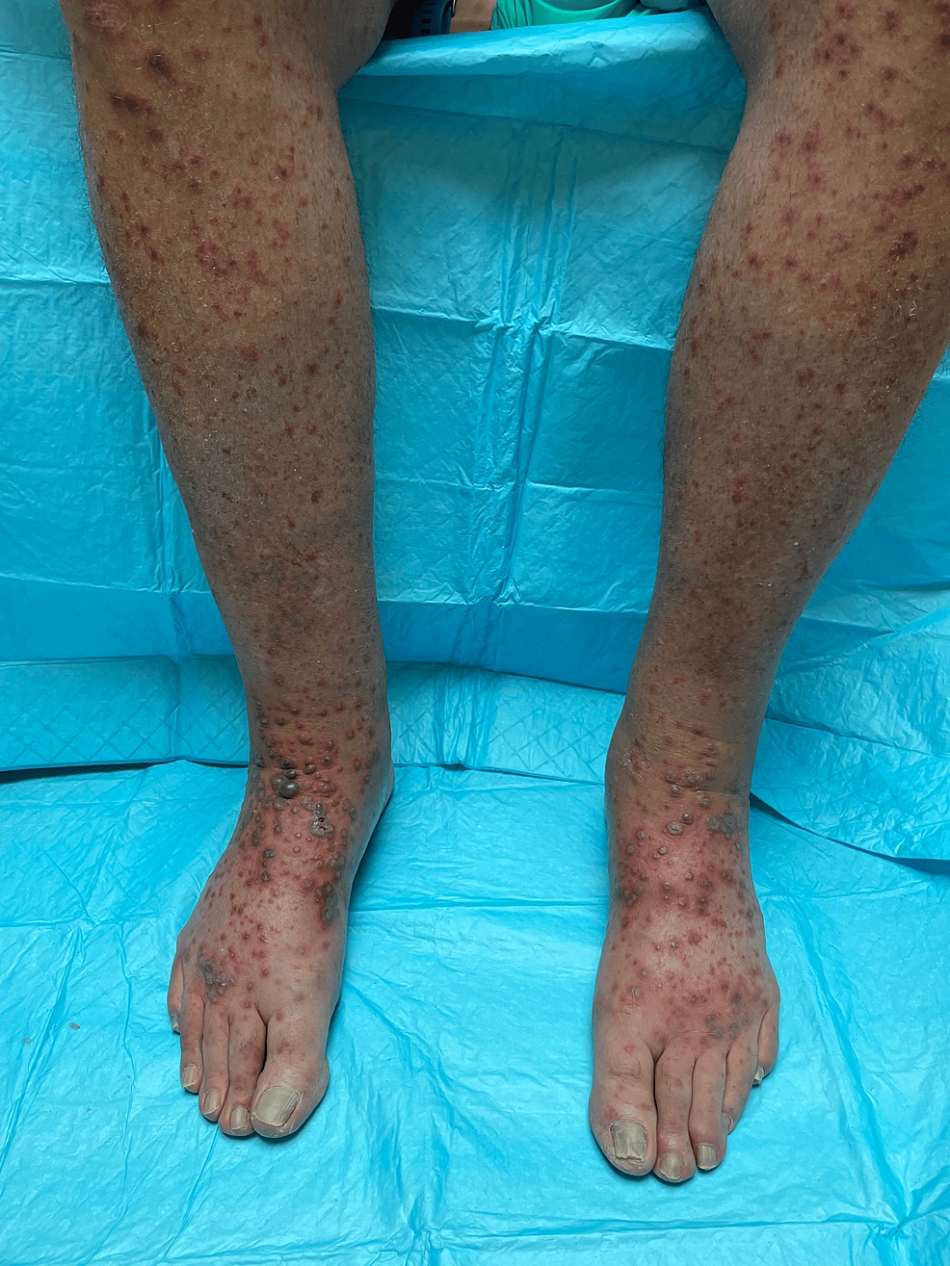
Image of the distal lower extremities and bilateral dorsal feet. Seen here are violaceous tense bullae overlying edematous targetoid papules coalescing into plaques.

**Figure 3:**
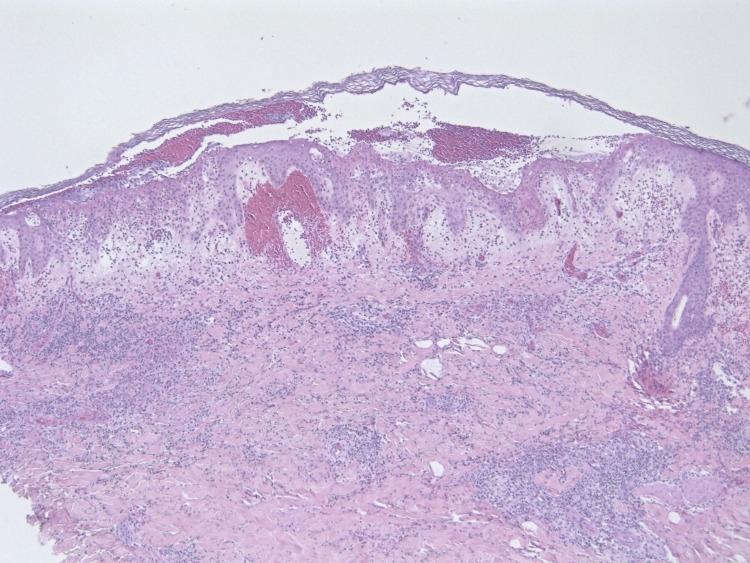
Superficial and deep inflammation with prominent subepidermal edema (magnification, 2×).

**Figure 4:**
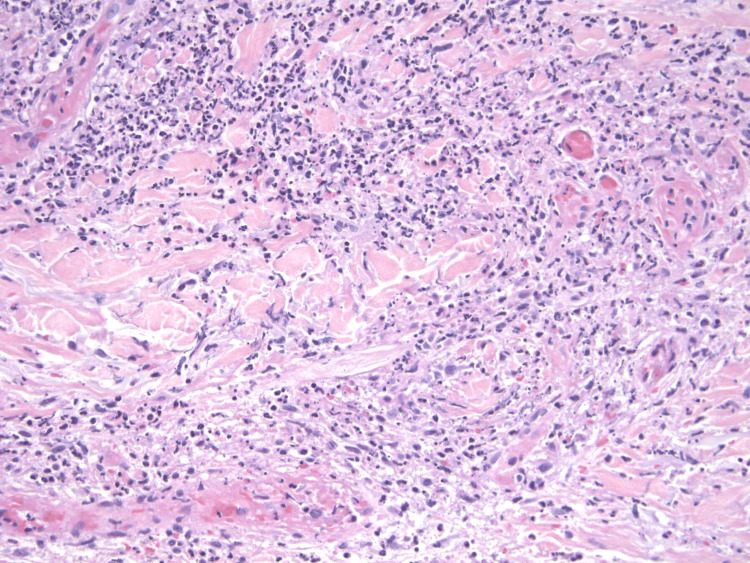
Neutrophilic predominant inflammation with leukocytoclasia and endothelial swelling but no vasculitis (magnification, 20×).

In this case, we present a 72-year-old male with a history of melanoma in situ, nonmelanoma skin cancer, hypertension, and coronary artery disease who presented to the clinic with a three-day history of a progressive bullous, erythematous papular rash that began on his right forearm and quickly spread to involve his extremities, trunk, palms, and soles. The rash was mildly pruritic but nontender. He had no history of recent febrile illnesses. His medications at the time included gabapentin, metoprolol tartrate, famotidine, loratadine, hydrochlorothiazide, and losartan. On inspection, there were multiple, small, edematous, violaceous plaques with overlying bullae at a few places (Figures 1, 2). Histopathologic analysis of punch biopsies obtained from lesions on his abdomen and thigh demonstrated dense inflammatory infiltrates of neutrophils, eosinophils, histiocytes, and lymphocytes, suggestive of a neutrophilic dermatosis, particularly Sweet’s syndrome (Figures 3, 4). He was treated with a prednisone taper of six weeks starting at 1 mg/kg/day with rapid improvement in his cutaneous symptoms, near resolution one month later. He was concomitantly treated with Domeboro soaks and clobetasol ointment for hands and feet to speed resolution. One month after presentation, he was started on fluocinonide twice a day for the residual rash on his feet. In addition to the management of his cutaneous symptoms, a malignancy workup was initiated. Blood work showed neutrophilia as well as elevated free kappa and lambda light chains, elevated lactate dehydrogenase (LDH), and elevated C-reactive protein (CRP) levels (Table 1). A positron emission tomography (PET) scan revealed lesions in the esophagus and kidney. Our patient was referred to Heme/Onc, GI, and Urology. He was diagnosed with esophageal adenocarcinoma stage IIb and bilateral probable simple and hemorrhagic renal cysts. As of this report, he has completed neoadjuvant chemotherapy and radiation for his esophageal cancer. He is s/p robotic Ivor-Lewis esophagectomy with no evidence of residual carcinoma on pathology and is undergoing surveillance with imaging every three months for his renal mass.

**Table 1:** Patient’s laboratory results showing elevated free kappa, free lambda, LDH, and CRP values at the time of malignancy workup. Also shown are reference values for the laboratory results. LDH: lactate dehydrogenase, CRP: C-reactive protein

Laboratory study	Results	Reference range
White blood cells	12.22 × 10^9^/L	3.90-9.90 × 10^9^/L
Absolute neutrophil count	10.53 × 10^9^/L	1.73-6.37 × 10^9^/L
Free kappa	32.98 mg/L	3.3-19.4 mg/L
Free lambda	34.44 mg/L	5.7-26.3 mg/L
LDH	322 U/L	105-233 U/L
CRP	6.7 mg/dL	0.8-1 mg/dL

## Discussion

Sweet’s syndrome has a characteristic constellation of fever, leukocytosis, and tender erythematous skin lesions with neutrophilic infiltrates on biopsy. This disease was first described in 1964 by Dr. Robert Douglas Sweet, who had documented these findings in eight separate female patients over the span of 15 years. Since then, we have achieved a broader understanding of this syndrome and the various associated extracutaneous manifestations, of which occult malignancy is the most concerning [4].

There are three main subsets of Sweet’s syndrome. Classic, or idiopathic, Sweet’s syndrome is characteristically seen in young females aged 30-50 years and seen in association with recent upper respiratory tract infection, inflammatory bowel disease, or pregnancy. The second subset, drug-induced Sweet’s syndrome, has been documented in various drugs including immunomodulatory drugs, antineoplastic agents, and antibiotics. The last subset, malignancy-associated Sweet’s syndrome (MASS), can manifest as a paraneoplastic syndrome in a known cancer patient or for an undiagnosed malignancy [5], as was the case with our patient. This demonstrates the importance for physicians to recognize the presentation of Sweet’s syndrome and understand its association with malignancy or as a sign of recurrence of malignancy. A retrospective longitudinal cohort study evaluating 52 patients diagnosed with Sweet’s syndrome from January 2000 to August 2020 found that 27 of the patients (51.9%) had MASS. Hematologic malignancies were more common than solid tumors in these patients. In those with solid tumors, cancers of the gastrointestinal (GI) tract were more common such as esophageal, colorectal, and gastric carcinomas [6].

Roughly half of patients with MASS present with classic cutaneous manifestations [4]. The presence of bullae is a rare variant of Sweet’s syndrome [7]. Rare and unique variants have been documented in cases of MASS, including bullous lesions, ulcerated lesions, and even lesions similar to pyoderma gangrenosum [4]. The literature reports a historical association with the presence of bullae and underlying hematologic malignancies. However, a retrospective study performed on 37 patients diagnosed with Sweet’s syndrome in a dermatology center in Singapore found that the presentation of bullous or pustular lesions was not exclusive to those with underlying hematologic malignancies [8]. Our patient presented with bullous lesions and an underlying solid tumor malignancy. Regardless of clinical presentation, Sweet’s syndrome warrants workup to rule out underlying malignancy.

Workup for MASS can be initiated with symptom-driven and age-appropriate screenings. Given the prevalence of hematologic malignancies, it is recommended that patients diagnosed with SS undergo a complete blood count (CBC) test with differential. Abnormalities in a CBC test should encourage consideration for a bone marrow biopsy in these patients. However, nonspecific peripheral leukocytosis with neutrophilia and an increase in erythrocyte sedimentation rate (ESR) and CRP are often seen in SS patients [9]. Our patient’s solid tumor malignancies were diagnosed using a PET scan. A high level of clinical suspicion for underlying malignancy should be maintained if no other explanation for SS can be elucidated.

Various hypotheses have been proposed for the pathogenesis of MASS. One theory proposes that Sweet’s syndrome is a hypersensitivity reaction to an antigen introduced by either a pathogen or a tumor. The basis of this theory stems from the clinical improvement in Sweet’s syndrome that occurs with corticosteroid treatment [4]. Patients with MASS who are not responsive to steroids initially will show improvement when the underlying malignancy is treated [6]. Notably, our patient experienced significant improvement in his cutaneous manifestations after the initiation of prednisone. Another theory proposes that MASS is the result of overproduction and dysregulation of various inflammatory cytokines [4].

## Conclusions

Underlying malignancies can present months to years after the diagnosis of Sweet’s syndrome. This makes the identification, workup, and active surveillance for underlying malignancy imperative for patients with Sweet’s syndrome. In this case report, we presented a patient diagnosed in a clinic with biopsy-proven Sweet’s syndrome, which improved with corticosteroid treatment, and further workup revealed underlying malignancy of the esophagus that had previously been asymptomatic. Swift identification and workup of this syndrome can greatly impact the life expectancies of these patients and save their lives, such as in this case. Therefore, this case emphasizes the impact that dermatologists can make in swiftly identifying MASS and getting these patients the potentially lifesaving treatment they need.
